# Oligomerization and characteristics of phosphoenolpyruvate carboxylase in *Synechococcus* PCC 7002

**DOI:** 10.1038/s41598-020-60249-2

**Published:** 2020-02-27

**Authors:** Claudia Durall, Sandesh Kanchugal P., Maria Selmer, Peter Lindblad

**Affiliations:** 1 Microbial Chemistry, Department of Chemistry - Ångström, Uppsala University, P.O. Box 523, SE-751 20 Uppsala, Sweden; 2 Department of Cell and Molecular Biology, BMC, Uppsala University, P.O. Box 596, SE-751 24 Uppsala, Sweden

**Keywords:** Biochemistry, Microbiology, Structural biology

## Abstract

Phosphoenolpyruvate carboxylase (PEPc) is an essential enzyme in plants. A photosynthetic form is present both as dimer and tetramer in C4 and CAM metabolism. Additionally, non-photosynthetic PEPcs are also present. The single, non-photosynthetic PEPc of the unicellular cyanobacterium *Synechococcus* PCC 7002 (*Synechococcus*), involved in the TCA cycle, was examined. Using size exclusion chromatography (SEC) and small angle X-ray scattering (SAXS), we observed that PEPc in *Synechococcus* exists as both a dimer and a tetramer. This is the first demonstration of two different oligomerization states of a non-photosynthetic PEPc. High concentration of Mg^2+^, the substrate PEP and a combination of low concentration of Mg^2+^ and HCO_3_^−^ induced the tetramer form of the carboxylase. Using SEC-SAXS analysis, we showed that the oligomerization state of the carboxylase is concentration dependent and that, among the available crystal structures of PEPc, the scattering profile of PEPc of *Synechococcus* agrees best with the structure of PEPc from *Escherichia coli*. In addition, the kinetics of the tetramer purified in presence of Mg^2+^ using SEC, and of the mixed population purified in presence of Mg^2+^ using a Strep-tagged column were examined. Moreover, the enzyme showed interesting allosteric regulation, being activated by succinate and inhibited by glutamine, and not affected by either malate, 2-oxoglutarate, aspartic acid or citric acid.

## Introduction

Phosphoenolpyruvate carboxylase (PEPc) is a carbon dioxide fixing enzyme that in an irreversible manner and in the presence of Mg^2+^, converts phosphoenolpyruvate and bicarbonate into oxaloacetate and inorganic phosphorus. It is present in bacteria (including cyanobacteria), algae, fungi and plants^1^. PEPc has been demonstrated to be involved in atmospheric CO_2_ fixation and storing carbon in cell vacuoles, play an anapleurotic role, supply energy for symbiotic bacteria, produce energy, abiotic stress acclimation, seed formation, and in the development and cell expansion^2^.

Plants have at least two PEPc enzymes, a plant-type PEPc and a bacterial-type PEPc. Plant-type PEPcs are categorized to be either photosynthetic (C4 or Crassulacean acid metabolism, CAM) or non-photosynthetic enzymes (C3 metabolism). Plant-type PEPcs are 105–110 kDa polypeptides with a conserved N-terminus and form a homotetrameric form, class 1. Plant bacterial-type PEPcs have larger polypeptides (116–118 kDa) with a non-conserved N-terminus, class 2. Class 2 polypeptides may associate with class 1 ones resulting in a hetero-octameric form^2^. In algae, PEPc exists in at least two isoforms; one being a homotetramer while the other(s) seems to consist of a carboxylase catalytic unit together with some unrelated polypeptides, whose proposed interactions may regulate the enzyme *in vivo*^3–6^.

In cyanobacteria, only one isoform of PEPc has been identified so far^7–9^. The cyanobacterial PEPc is present as a single copy gene and it is essential for the cells^10^. High order cyanobacteria possess PEPc amino acid sequences more similar to PEPc in C4 type higher plants, while in low order cyanobacteria the PEPc amino acid sequences do not resemble neither the PEPcs of C3 nor C4 plants^11^. In general, the PEPc enzymes have a conserved C-terminus and conserved essential amino acids for catalysis but differ in their N-terminus resulting in different regulations. For instance, plant PEPc enzymes have a conserved serine that can be phosphorylated thereby activating the enzyme^12–14^.

In the plant C3 metabolism, Ribulose- 1,5-bisphosphate carboxylase/oxygenase (RuBisCO) is the primary CO_2_ fixing enzyme^15–17^. In addition to fix CO_2,_ RuBisCO can function as an oxygenase where O_2_ is fixed and further metabolized in the so called photorespiration, an essential metabolic process in which CO_2_ is released^18^. PEPc in C3 plants, bacteria and cyanobacteria, contributes to the tricarboxylic acid cycle (TCA) playing an anapleurotic role providing carbon skeletons to the nitrogen metabolism which, at least in cyanobacteria, may account up to 20% of the total carbon fixed^8,10^.

In C4 and CAM plants, with a more efficient CO_2_ fixation process in which RuBisCO avoids to use O_2_ as a substrate, photorespiration is more or less negligible. PEPc, the primary carbon fixation enzyme, is present in the mesophyll cells in the leaves where it fixes CO_2_ and uses phosphoenolpyruvate (PEP) to produce oxaloacetate that is converted into malate. Malate is transported to the sheath cells, where RuBisCO is located, and is converted to pyruvate and CO_2_. Evolved CO_2_ is fixed by the most abundant protein on earth, RuBiSCO. Pyruvate is finally transported to the mesophyll cells in order to restart the cycle^19^.

It is known that C4 plants fix carbon through PEPc and RuBisCO during conditions of light and RuBisCO needs significant amounts of NADPH and ATP produced by the light dependent photosynthetic reactions. By contrast, CAM plants close the stomata during the day in order to avoid evaporation of water and CO_2_ cannot be fixed by PEPc. In addition, malate is present in the cytoplasm of the cells and any free metal ions are complexed. These factors dissociate the carboxylase to a dimer, which is the inactive form. During darkness, the stomata are open, divalent cations are free, malate concentration is low and the oligomerization state of the enzyme is a tetramer which is the active form. As consequence, PEPc fixes CO_2_ and the plant stores malate in the vacuole until the light reactions of photosynthesis are active again and RuBisCO can fix the CO_2_ released from malate by the malic enzyme^20^.

Different studies have examined the activity and regulation of PEPc in selected cyanobacterial strains^7–9^. However, so far, there are no studies addressing the oligomerization states of PEPc in cyanobacteria and this an important factor to understand the regulation of this enzyme *in vivo*. Herein, we examine and characterize the single PEPc from the low order unicellular cyanobacterium *Synechococcus* PCC 7002. The faster growth of this organism compared to e.g. *Synechocystis* PCC 6803^21^ may indicate different kinetics of the CO_2_ fixing enzymes and despite being a marine strain has conserved amino acids residues characteristic of PEPc from freshwater strains^14^.

## Results

### Expression and purification of PEPc PCC 7002

**Figure 1:**
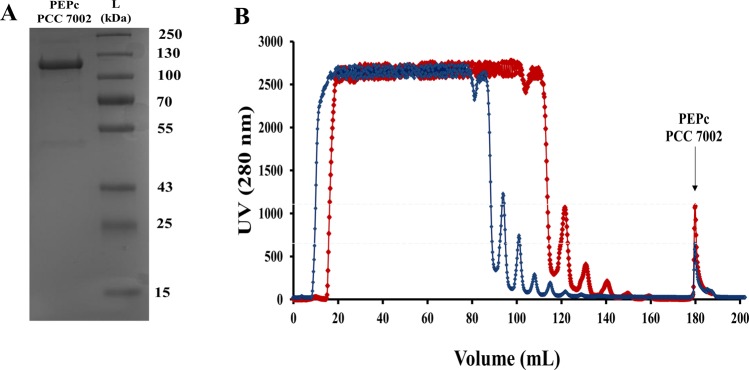
Purification of PEPc from the cyanobacterium *Synechococcus* PCC 7002. (**A**) Purified PEPc from the cyanobacterium *Synechococcus* PCC 7002. PEPc PCC 7002 corresponds to the purified PEPc with a tag attached to the N-terminus. L corresponds to the ladder used in kDa. (**B**) Chromatogram showing the purification of PEPc PCC 7002 using a Strep-Column and chromatography with TBS pH 8.0 (Blue) or TBS pH 8.0 and 25 mM MgCl_2_ (Red).

PEPc PCC 7002 was cloned with a Strep-tag and expressed and harvested in *Escherichia coli* in order to be purified. After trying different conditions, the optimal IPTG concentration and temperature for expression of PEPc from *Synechococcus* PCC 7002 (*Synechococcus*) were 0.1 mM IPTG and 25 °C, respectively. Interestingly, when the Strep tag was attached to the C-terminus of the PEPc from *Synechococcus*, the protein did not attach to the column and no enzyme could be purified (data not shown). However, changing the position of the Strep tag to the N-terminus of the carboxylase resulted in a successful purification of PEPc PCC 7002 with a size agreeing with the theoretical molecular weight of 115.4 kDa (Fig. 1A). In addition, when the carboxylase was purified in the presence of Mg^2+^ (red line) the amount of purified protein increased 2-fold compared to in absence of the divalent cation (blue line) (Fig. 1B).

### Oligomerization of PEPc PCC 7002

**Figure 2:**
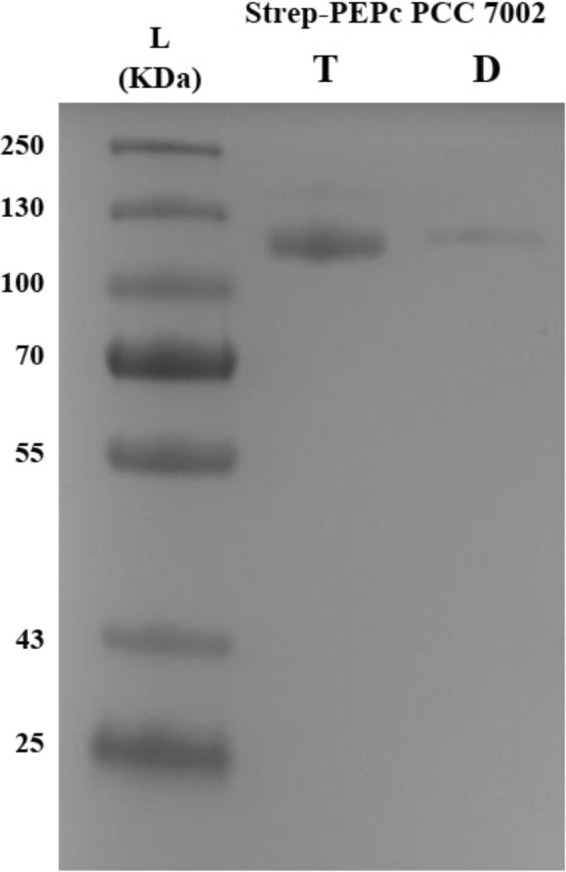
SDS-PAGE showing the eluted PEPc PCC 7002 after the PEPc reaction was performed (Mg_10+ HCO_3_^−^+PEP, Table 1). D corresponds to the dimer form of PEPc PCC 7002, T corresponds to the tetramer form of PEPc PCC 7002, and L corresponds to the ladder used in kDa.

The purified PEPc PCC 7002 was analysed using size exclusion chromatography (SEC). Using the Superdex 200 column, a peak corresponding to a low molecular weight oligomer of PEPc PCC 7002 (D_PEPc PCC 7002) was present in all purification conditions examined (Dimer, Table 1). Additionally, another higher molecular weight oligomer (larger oligomer) peak (T_PEPc PCC 7002) was present under few conditions (Tetramer, Table 1). High concentration of Mg^2+^ (Mg_25, Table 1, Supplemenary Information Fig. S1) induced the larger oligomer but lower concentration of the divalent cation did not (Mg_10, Table 1). When HCO_3_^−^ was used, the larger oligomer was not present (HCO_3_^−^, Table 1). Interestingly, when HCO_3_^−^ and the lower concentration of Mg^2+^ were combined, the larger oligomer was eluted (Mg_10+ HCO_3_^−^, Table 1). Presence of PEP induced the larger oligomer when used alone, or combined with either HCO_3_^−^ or low concentration of Mg^2+^ (PEP, HCO_3_^−^+ PEP and Mg_10+ PEP, Table 1). When an *in vitro* reaction was performed^22^, both oligomers were eluted (PEP + HCO_3_^−^ + Mg_10, Table 1, Fig. 2).

**Table 1:** Oligomerization forms of PEPc from *Synechococcus* PCC 7002 when eluted from the Superdex (SEC) using TBS buffer with different additives.

Buffer	Addition to the TBS buffer	Tetramer	Dimer	Ratio (Tetramer:Dimer)	Supplementary Fig. S1
TBS	−	−	+	0:1	A
Mg_25	25 mM MgCl_2_	+	+	1.4:1	B
Mg_10	10 mM MgCl_2_	−	+	0:1	C
HCO_3_^−^	5 mM NaHCO_3_^−^	−	+	0:1	D
PEP	5 mM PEP	+	+	1.1:1	E
Mg_10+ HCO_3_^−^	10 mM MgCl_2_ and 5 mM NaHCO_3_^−^	+	+	0.8:1	F
Mg_10+PEP	10 mM MgCl_2_ and 5 mM PEP	+	+	1.3:1	G
HCO_3_^−^+ PEP	5 mM NaHCO_3_^−^ and 5 mM PEP	+	+	0.8:1	H
Mg_10+ HCO_3_^−^+PEP	10 mM MgCl_2,_5 mM NaHCO_3_^−^ and 5 mM PEP	+	+	unknown	I

### Small angle X-ray scattering (SAXS)

**Figure 3:**
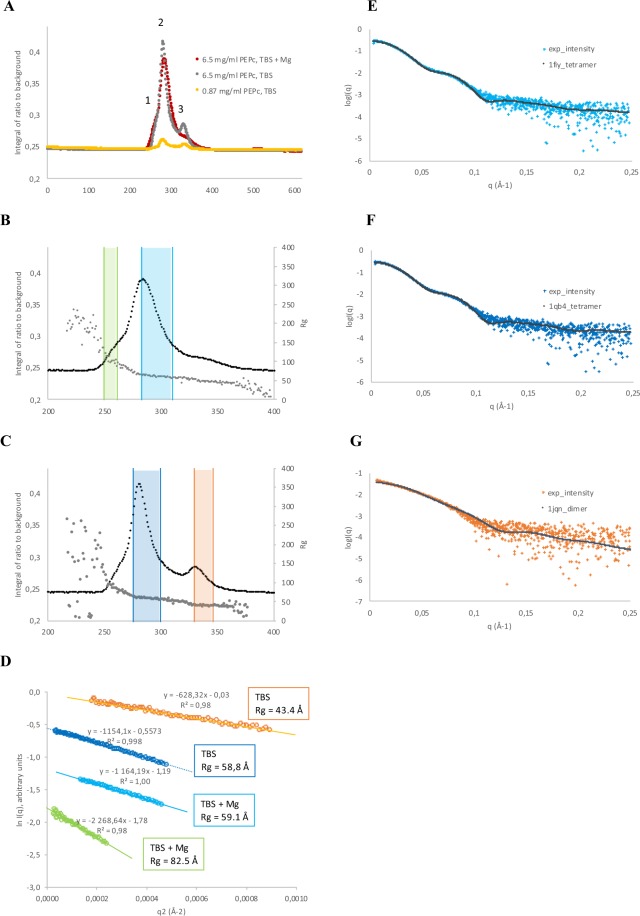
SEC-SAXS data. (**A**) Overlay of signal plots from SEC-SAXS of PEPc in TBS at two different concentrations (yellow, grey) and in TBS with 25 mM MgCl_2_. Peaks numbers are indicated. (**B**) Zoom in of signal plot for 6.5 mg.ml^−1^ PEPc in TBS with 25 mM MgCl_2_ (red). Grey markers indicate the radius of gyration calculated from the individual scattering curves. Green bars indicate the data frames used for analysis of peak 1, cyan bars indicate the data frames used for analysis of peak 2. (**C**) Zoom in of signal plot for 6.5 mg · ml^−1^ PEP c in TBS. Grey markers indicate the radius of gyration calculated from the individual scattering curves. Blue bars indicate the data frames used for analysis of peak 2, orange bars indicate the data frames used for analysis of peak 3. (**D**) Guinier plots used to derive R_g_ for peak 1 and 2 in TBS + Mg (bottom) and for peak 2 and 3 in TBS (top). Colors as in B–C. (**E**) Experimental scattering curve for peak 2 in TBS + Mg overlaid with calculated scattering curves from a tetramer generated from PDB entry 1fiy. (**F**) Experimental scattering curve for peak 2 in TBS overlaid with calculated scattering curves from a tetramer generated from PDB entry 1qb4. (**G**) Experimental scattering curve for peak 2 in TBS overlaid with calculated scattering curves from a dimer generated from PDB entry 1jqn.

In order to further clarify and confirm which oligomeric states of PEPc from *Synechococcus* were present at different conditions, size-exclusion chromatography coupled to small-angle X-ray scattering (SEC-SAXS) was performed at beamline B21 at Diamond Light Source. At all tested conditions, three peaks could be observed, an initial shoulder (peak 1), a main peak (peak 2) and a smaller peak or shoulder at the end (peak 3) (Fig. 3A). Using the estimated radius of gyration (R_g_) for each data frame as a guide (Fig. 3B,C), scattering curves from each peak were analyzed separately to determine the size and shape of the different oligomers in the sample.

The radii of gyration and molecular weights derived using the different available methods (Fig. 3D, Table 2) show reasonable agreement with the minor peak 1 consisting of octamers (theoretical MW 924 kDa) or decamers (theoretical MW 1.15 MDa), the main peak 2 containing tetramers (theoretical MW 462 kDa) and peak 3 containing dimers (theoretical MW 231 kDa) (Table 2). The Kratky plots for peaks 1 and 2 shows the double bell-shape characteristic of multidomain proteins, while the corresponding plot for peak 3 shows a normal bell-shape indicating that the dimer is compact (Supplementary Information, Fig. S2). Based on the Kratky plots (Supplementary Information, Fig. S2), all oligomers are well folded.

**Table 2:** Molecular parameters derived from analyses of SEC-SAXS (Fig. 3A–D) and from batch SAXS (Fig. 4) data.

Buffer	SEC-SAXS peak	R_g_ (Å) from Primus^38^	Baysean inference MW (kD) (MW probability (%))^39^	MW (kD) from volume of correlation	Rg (Å) from SAXSMoW	MW from SAXSMoW^41^
TBS + Mg	1	82.5	873 (92.3)	1119	82.7	1133
TBS + Mg	2	59.1	479 (79.0)	510	59.8	524.5
TBS	2	58.8	434 (70.6)	465	58.7	492.5
TBS	3	43.4	170 (81.3)	157	43.0	174.6
**Buffer**	**Batch sample conc. (mg/ml)**	**R**_**g**_ **(Å)**	Baysean inference MW (kD) (MW probability (%))^*^	**MW (kD) from volume of correlation**	**Rg (Å) from SAXSMoW**	**MW from SAXSMoW**
TBS	0.2	57.5	243 (76.3)	252	56.8	318.2
TBS	0.5	58.6	318 (99.9)	330	58.2	422.5
TBS	1.0	62.3	392 (40.2)	430	62.0	486.9
TBS	3.0	66.5	479 (90.8)	500	66.1	560.5
TBS	4.3	68.3	479 (95.0)	523	68.2	589.5

Comparison of the SEC-SAXS signal plots for PEPc at 6.5 and 0.87 mg · ml^−1^ in TBS (Fig. 3A) shows that the proportion of dimer increases at lower concentration, consistent with a concentration-dependent equilibrium between dimer and tetramer. The signal plot in presence of 25 mM Mg^2+^ shows a smaller fraction of dimer than in absence of Mg^2+^, suggesting that the tetramer is stabilized by Mg^2+^.

There are available crystal structures of PEPc from bacteria (*E. coli*) and plants (maize, *Flaveria trinervia and F. pringlei*), but there is at present no structure of a PEPc from any cyanobacterium. Aiming to find out which of the available crystal structures were most similar to PEPc PCC 7002, the experimental scattering curves were compared to calculated scattering curves of biological dimers and tetramers generated from the available PDB entries (Supplementary Information, Table S1). The tetramer observed in solution is most similar to the *E. coli* PEPc tetramers (Fig. 3E,F, PDB entries 1FIY, 1JQN, 1QB4). Similarly, the dimer observed in solution is most similar to the *E. coli* PEPc dimers (Fig. 3G, PDB entries 1FIY, 1JQN, 1QB4). The dimer data shows a better fit with calculated scattering curves (lowest χ^2^ 1.47) than the tetramer data (lowest χ^2^ 5.03), suggesting that some changes compared to the crystallized tetramer are observed in solution. The crystal packing in the available structures does not suggest any obvious octamer or decamer, for which reason no fitting was attempted for peak 1.

**Figure 4:**
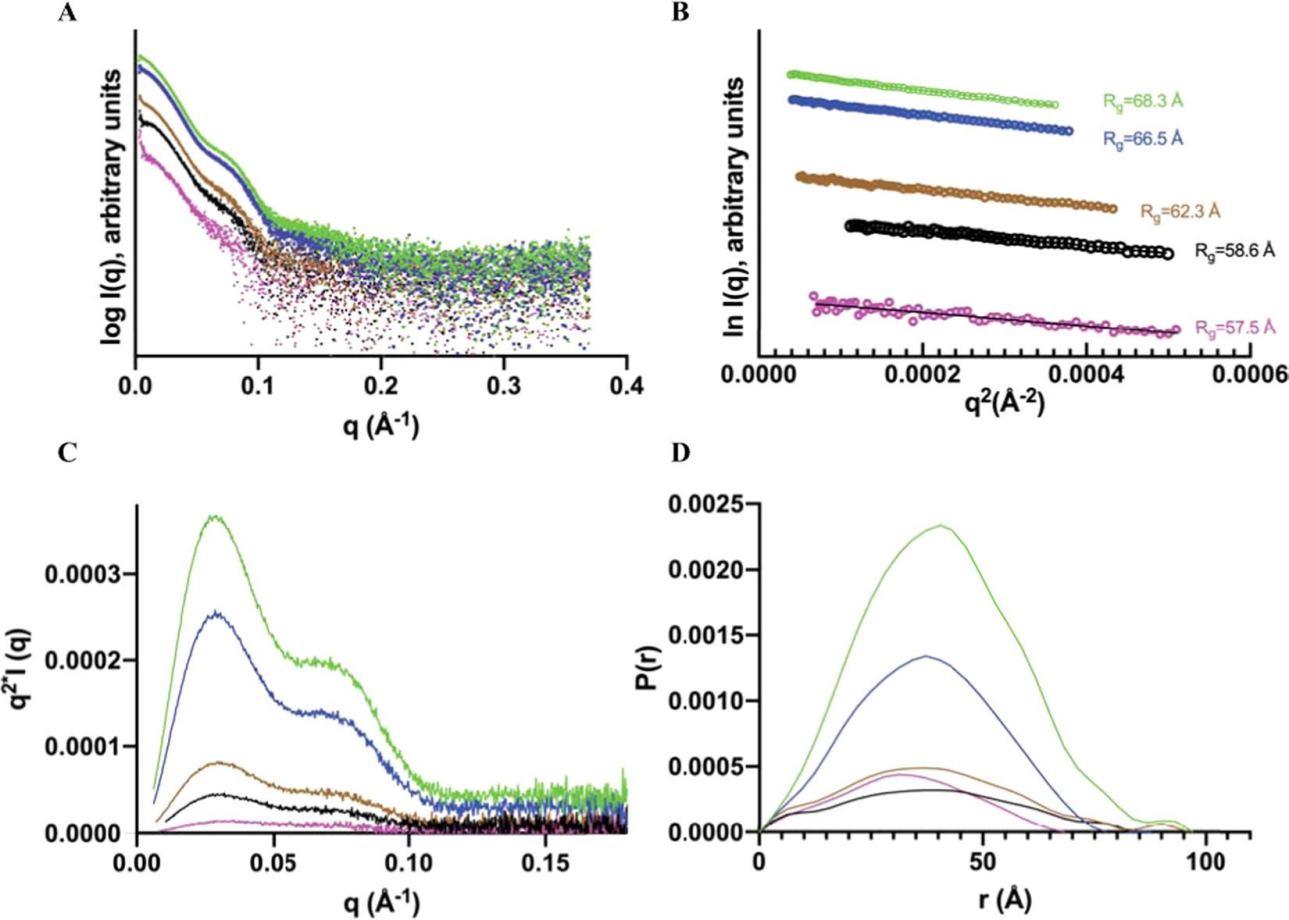
Batch SAXS data and analysis, all data have been plotted using offset for visualization purposes. (**A**) Experimental scattering curve of PEPc in TBS buffer at different concentrations (green 4.3 mg ^.^ ml^−1^, blue 3 mg ^.^ ml^−1^, brown 1 mg ^.^ ml^−1^, black 0.5 mg ^.^ ml^−1^ and magenta 0.2 mg ^.^ ml^−1^). (**B**) Guinier plots used to derive R_g_ values, colors are as in A. (**C**) Kratky plots, colors as in A. (**D**) Distance distribution P(r) plots. Derived D_max_ values are 97, 97, 95, 83 and 80 Å.

The SAXS experiments were also performed in batch mode in TBS buffer at different protein concentrations. The shape of the scattering curve changes with protein concentration (Fig. 4A), in agreement with a concentration-dependent equilibrium. Guinier analysis (Fig. 4B) and estimation of average molecular weight shows that the average oligomer size increases with concentration (Table 2) which can also be observed in the Kratky and P(r) plots (Fig. 4C,D). However, even at the lowest tested concentration, 0.2 mg · ml^−1^, the estimated R_g_ and molecular weight are higher than for the dimer in peak 3 of the SEC-SAXS analysis, showing that PEPc never completely dissociates to dimers under these experimental conditions. Importantly, these measurements would be skewed towards larger sizes if any fraction of aggregates were present in the samples. At the lowest concentrations, the batch data is relatively noisy and may show signs of aggregation (Fig. 4A), but there is no sign of aggregation at the higher concentrations or in the SEC-SAXS data (Fig. 3A).

### Kinetic characterization of PEPc *Synechococcus* PCC 7002

**Figure 5:**
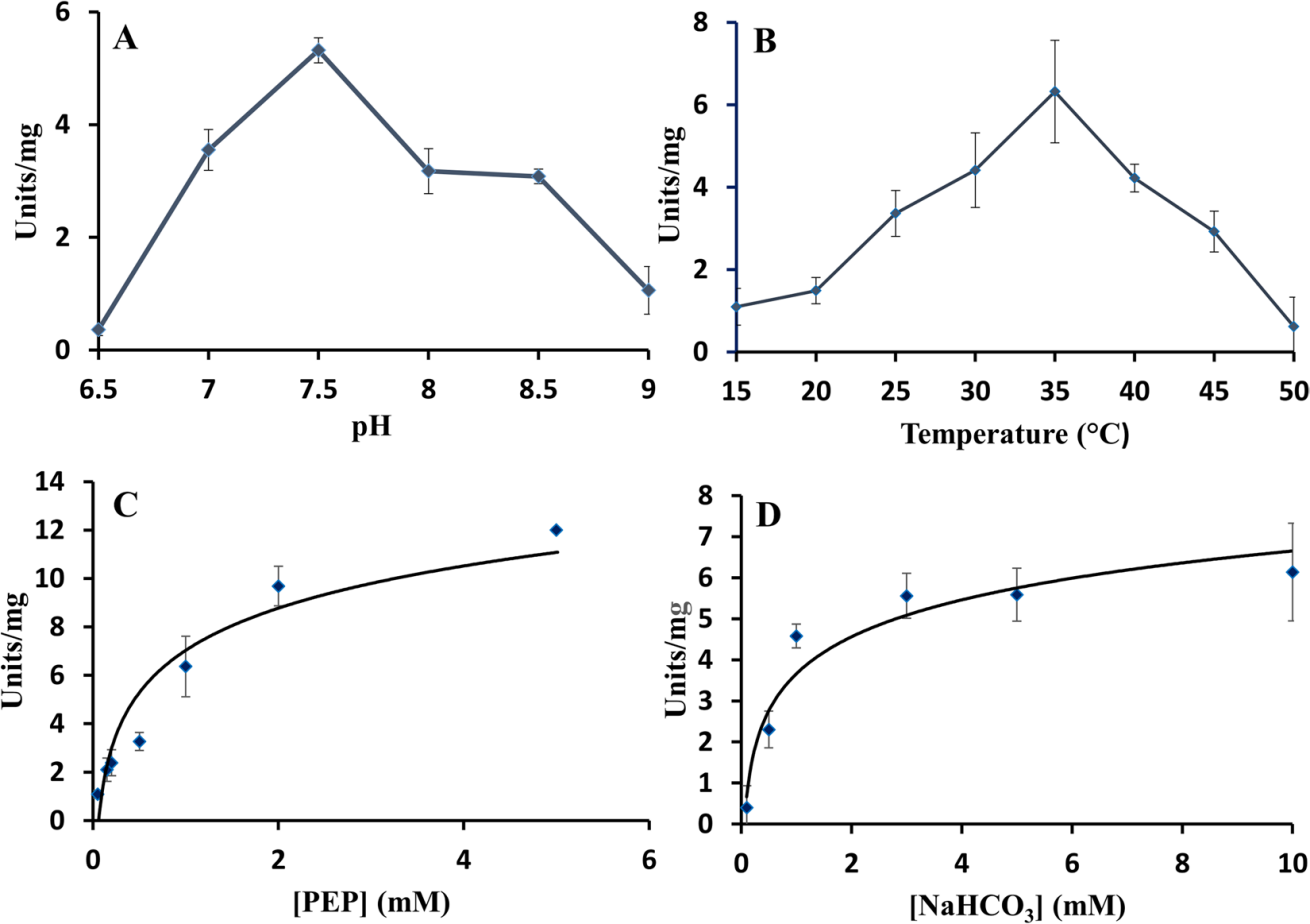
Specific activity of purified PEPc PCC 7002 (Table 1). (**A**) Specific activity of PEPc PCC 7002 with different pHs (23 °C). (**B**) Specific activity of PEPc PCC 7002 with different temperatures (pH 7.5). (**C**) Specific activity of PEPc PCC 7002 dependent on the concentration of PEP (pH 7.5, 35 °C). (**D**) Specific activity of PEPc PCC 7002 dependent on the concentration of NaHCO_3_ (pH 7.5 35 °C). One unit is defined as 1 mol of NAD^+^ produced per minute.

**Figure 6:**
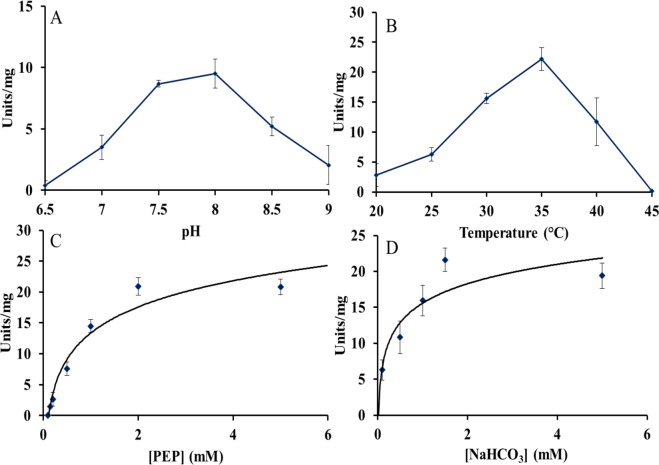
Specific activity of the T_PEPc PCC 7002- tetramer eluted with 25 mM MgCl_2_ (Supplementary Information Fig. S1-B, Table 1). (**A**) Specific activity at different pH (23 °C). (**B**) Specific activity of at different temperatures (pH 8.0). (**C**) Specific activity as function of the concentration of PEP (pH 8.0, 35 °C). (**D**) Specific activity as function of the concentration of NaHCO_3_ (pH 8.0, 35 °C). One unit is defined as 1 µmol of NAD^+^ produced per minute.

**Figure 7:**
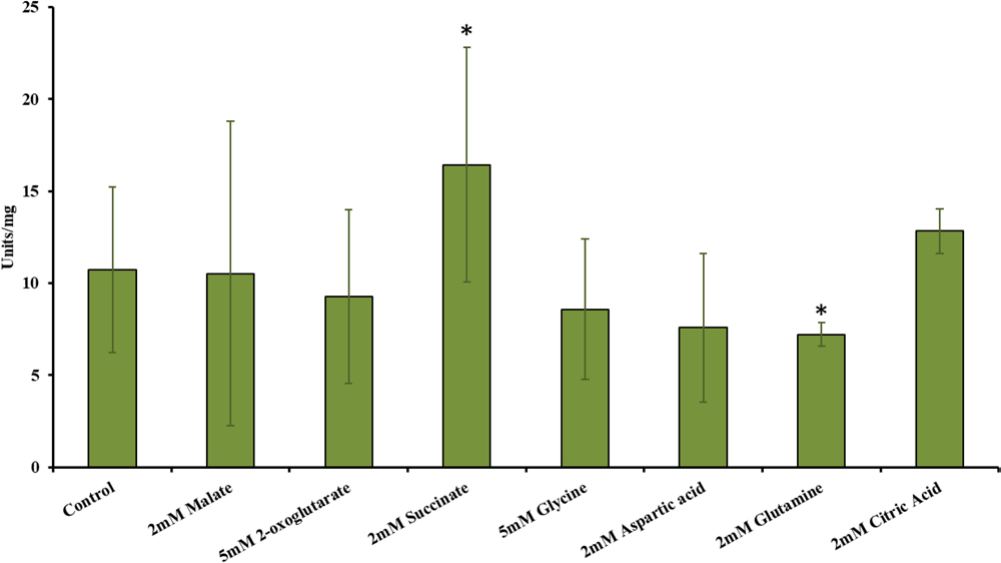
Specific activity of T_PEPc PCC 7002 in the presence of potential inhibitors or activators. The concentration of PEP and NaHCO_3_ was 1 mM. Asterisks asterisk indicates statistically significant difference (T-Test) compared to the control.

Purified PEPc PCC 7002 and T_PEPc PCC 7002 were characterized. The optimal pH of PEPc PCC 7002 was determined to be 7.5 for the purified protein with Mg^2+^ and 8.0 for T_PEPc PCC 7002, even though no significant differences were observed between pH 7.5 and 8.0 (Figs. 5A and 6A, respectively). The optimal temperature was 35 °C (Figs. 5B and 6B). The enzyme was found to be unstable with time, but it clearly obeyed Michaelis-Menten kinetics (Figs. 5, 6C,D). Calculated V_max_ for the PEPc PCC 7002 was 14.4 ± 2.5 units·mg^−1^ and the T_PEPc PCC 7002 20.7 ± 1.8 units·mg^−1^. The K_m_ for PEP and HCO_3_^−^ were 1.06 and 0.97 mM for PEPc PCC 7002, and 0.77 and 0.24 mM for T_PEPc PCC 7002, respectively. The D_PEPc PCC 7002 eluted using Mg^2+^ (Mg_25, Table 1) showed a V_max_ of 9.54 ± 1.1 units·mg^−1^. Interestingly, neither malate, 2-oxoglutarate, glycine, aspartic acid or citric acid, potential inhibitors or activators of PEPc, did affect the V_max_ of the respective reaction (Fig. 7). However, addition of either succinic acid or glutamine resulted in significant differences in the observed V_max_ (p = 0.043 and p = 0.037, respectively) (Fig. 7). Thus, succinic acid and glutamine were shown to be an activator and an inhibitor, respectively, of T_PEPc PCC 7002.

## Discussion

Phosphoenolpyruvate carboxylase (PEPc) of *Synechococcus* PCC 7002 was examined with the aim to examine oligomerization, structure and characteristics. The activity of PEPc in C4 and CAM plants have shown to be regulated through the oligomerization states during light and darkness^23^. In addition, some compounds inhibit or activate a specific oligomer form of the carboxylase^23^. However, this has not addressed for any cyanobacterial PEPc. In order to purify the PEPc PCC 7002 a strep tag was added to the N-terminus, in agreement with a previous study^9^. When initially added to the C-terminus, we were unable to observe any binding to the column and no protein could be purified. This is probably a consequence of the C-terminus being buried inside the PEPc monomer, as is the case in homologous PEPc structures from bacteria and plants^24,25^. PEPc PCC 7002 purified under different conditions showed different oligomeric states. Using a high concentration of the cation Mg^2+^, a cofactor of PEPc, induced the tetramer form (Table 1, Fig. 3A), in agreement with earlier observations with PEPc from the plant *Crassula argentea*^20^. The tetrameric form may also have higher affinity to the Strep-column since four Strep tags are exposed compared to the dimeric form. It is also known that the tetramer is in all PEPcs stabilized by a salt bridge between arginine in position 503 and glutamic acid in position 498^24^ (PEPc from *Synechococcus*).When the protein is diluted, the fraction of dimer increases (Fig. 4, 25). This study showed two oligomerization states of the carboxylase based on the substrates and/or cofactor present. In addition, this is the first report showing two oligomeric states for a bacterial or cyanobacterial PEPc^7–9,24^.

The differences observed in the ratio of the two peaks in the two SEC columns used (Superdex and Shodex, Table 2 and Fig. 3A) can be attributed to the fact that the columns have different volumes (100 and 4.6 mL, respectively), resulting in different degrees of dilution and different running times that may or may not allow the tetramer to reach equilibrium and dissociate into dimers during the run. In contrast to what has been shown for PEPc from *C. argentea*^20^, a pure tetramer was never observed (Table 2 and Fig. 3A). In addition, we also observed a small fraction of a larger oligomer (octamer or possibly decamer) in all SEC-SAXS runs. This has never been observed before, and requires high-resolution SEC to be detected, but a hetero-octamer has been suggested for PEPc from class 2^2^. Remarkably, we identified substrates of PEPc which induce the tetramer form (Table 1). The concentrations of the substrates used for the SEC are based on the concentrations used in our previous activity assays^26^.

It has been suggested that the biphasic behaviour of the enzyme when Mg^2+^ is present is due to a modification of a histidine residue^26^. Mukerji^27^ suggested that Mg^2+^ is an activator of PEPc. The fact that Mg^2+^ can be an activator may influence the oligomerization state of protein but the process is still unclear. In the present study, we observed that Mg^2+^ influences the oligomerization state of PEPc PCC 7002 (Table 1, Supplementary Information Figs. S1-B and 3). In addition, the low concentration of Mg^2+^ and HCO_3_^−^ or PEP has shown to induce the tetramer oligomer of PEPc PCC 7002 and this a novel result (Table 1). However, it has been observed that the Mg^2+^-PEP complex may induce the tetramerization form in maize PEPc^28^. Our experiments show that the tetramer is the main oligomer after the reaction (Fig. 2), in agreement with other studies that have shown that the tetramer of PEPc is the active form of the protein^20^.

Since the PEPc PCC 7002 showed different activity levels and the exact values dropped by half upon dilution (Fig. 5), the tetrameric form of PEPc PCC 7002 from purification in presence of Mg^2+^ was also characterized kinetically. The observed optimal pH of the PEPc PCC 7002 was in the same range as for all cyanobacterial PEPcs characterized so far (Table 3) and in full agreement with PEPc in *Anabaena* PCC 7120^9^. The activation energy of T-PEPc PCC 7002 was estimated to be 59.3 KJ/mol, more than double of PEPc from the cyanobacterium *Coccochloris peniocystis*^8^. According to our results, PEPc PCC 7002 showed Michaelis-Menten kinetics for both substrates, similar as for PEPc from *C. peniocystis*^8^ and contrary to PEPc from the green alga *Selenastrum minutum*^3^.

**Table 3:** Characteristics of PEPc from different organisms.

Organism	T (°C)	pH	V max (units/mg)	Km (PEP) mM	Km (HCO_3_^−^) mM	Inhibitor/Activator	Reference
*Anabaena* PCC 7120 (filamentous cyanobacterium)	35	8	2.6	1.1	0.24	A, M	^9^
*Coccochloris peniocystis* (unicellular cyanobacterium)	40	8	8.84	0.6	0.8	O, M lesser extent: C, IC, O, ATP, D, ML, P/Pyr, 3PGA, NADPH	^8^
*Synechococcus* PCC 7002 (unicellular cyanobacterium) tetramer form	35–35	7.5–8	14.43–20.74	1.06–0.77	0.97–0.24	n.t.-Q/S	This study
*Synechococcus vulcanus* (unicellular cyanobacterium)	42* (30)	9 7.5	25.3 17.3	0.53 0.58	nd 0.48	D	^7^
*Synechocystis* PCC 6803 (unicellular cyanobacterium)	30	7.3	1.74	0.34	0.8	Weak inhibition: S, M, F, C (pH 7.3) Strong inhibition: M, A (pH = 9)	^9^
*Oceanimonas smirnovii* (marine bacterium)	20 (RT)	10	21.8	1.22	0.139		^30^
*Chlamydomonas reinhardtii* (green alga)	25 (n.s)	8.8 8.1	22 18			E,D,O,M/Q, DAP	^5^
*Selenastrum minutum* (green alga)	25 (n.s)	9 9	5.29 5.71	(S50) 2.23 0.32		Q	^3^
*Zea mays* (C4 plant)	30 (n.s)	7.3 8	18.2 23	1.48 0.59	0.12 0.1	M, D/G6P	^7,9,23^

Abbreviations: ACoa- Acetyl-CoA, ATP- Adenosine Triphosphate, C-Citrate, D-Aspartate, DAP- Dihydroxyacetone phosphate, E-Glutamate, F- Fumarate, G6P- Glucose-6-Phosphate, IC-Isocitrate, M-Malate, ML- Manolate, NADPH-Nicotinamide adenine dinucleotide phosphate, OAA-Oxaloacetate, O-2 oxoglutarate, P-Phosphate, Pyr- Pyruvate, Q-Glutamine, RT- Room temperature, S-succinate, 3PGA- 3-phosphoglyceraldehyde, (n.s)- temperature used for the activity assay but optimal temperature not specified, *Optimal temperature, in parentheses temperature used for the activity assay, n.t. no tested.

The PEPc PCC 7002 has the highest Km for HCO_3_^−^ and PEP compared to other cyanobacterial PEPcs (Table 3)^29^. The fact that the T_PEPc PCC 7002 has a higher affinity for HCO_3_^−^ compared to the PEPc PCC 7002 can be due to T_PEPc PCC 7002 may be mainly tetramers (active form) while PEPc PCC 7002 might be an equilibrium between tetramers and dimers. In addition, in agreement with Takeya *et al*.^9^ and Schylajanaciyar *et al*.^11^, it seems that strains belonging to Chroococcales have a lower Km for PEP than those in Nostocales, as PEPc from *Anabaena* PCC 7210 showed an even higher Km value for PEP than PEPc PCC 7002 (Table 3). However, we believe that in the cyanobacterial cells, PEP is not a limiting metabolite. In light, when photosynthesis is active and RuBisCO fixes CO_2_^18^, and in darkness, when glycolysis is active, the level is lower but there is still PEP in the cells^30,31^. It is relevant to note that cyanobacteria have active inorganic carbon transporters^32^ and that the amount of bicarbonate inside the cells is around 4 mM during the day and lower during the night^33^ but PEPc is also active in darkness (35, unpublished) and as a consequence the carboxylase may not necessarily have high affinities for these substrates.

Malate is a well-known inhibitor of PEPc^7,9,20,34^. However, under the conditions examined, the T_PEPc PCC 7002 activity was not repressed by malate and this is in agreement with observations for PEPc from the cyanobacterium *S. volcanus* (at pH 7.5)^7^. In plants, the photosynthetic PEPc seems to be regulated by malate and other factors. Firstly, PEPc of C3 plants, *Mesembrutanthermum crystallinun* and *Spinacia oleracea*, from light and dark forms, are inhibited by malate^34^. Secondly, C4 PEPc from *Zea mays* showed higher sensitivity to malate in darkness, which is typical for C4 plants^33^. Thirdly, in the CAM plant *Crassula* malate inhibits PEPc in the dimeric form and it might be responsible to regulate the carboxylase activity during day and night cycles^20,34^. This study can conclude that malate is not an allosteric inhibitor of T_PEPc PCC 7002 when Mg^2+^ is present, but it might be an inhibitor when Mg^2+^ is absent or induce dissociation into dimers in absence of divalent ions. Interestingly, high order cyanobacteria seem to have a conserved serine residue characteristic for PEPc of C4 plants, an amino acid suggested to be involved in aspartate inhibition^9,11^. Conversely, PEPc of some low order cyanobacteria (e.g. *Synechocysti*s PCC 6803 and *Synechococcus* PCC 7002) have a serine residue in the corresponding position (S817 in *Synechococcus*) but do not show any inhibition by aspartate (9, this study). Succinate has been reported to be an inhibitor of PEPc^35,36^, but in T_PEPc PCC 7002, it was instead shown to be an activator (Fig. 7). Even though it is not fully clarified, it seems that succinate is also an activator for PEPc from *Synechocystis* PCC 6803^9^. Surprisingly, T_PEPc PCC 7002 was inhibited by glutamine, although glutamine has been reported to be an activator of PEPc in the green alga *Chlamydomonas reinhardtii*, in one isoform of PEPc in *S. minutum* as well as the close related cyanobacterium *S. volcanus*^3,5,7^. In addition, it has been shown to induce *pepc* expression in maize leafs^37^. According to our understanding, glutamine may be an activator of PEPc since overexpression of PEPc should lead to an increased 2-oxoglutarate level. 2-oxoglutarate and glutamine react in order to form glutamate. However, the limitation of this study is that in the *in vitro* assay, the protein is isolated under optimal conditions and this may not be the condition *in vivo*. Thus, it may be that *in vivo* there are other factors affecting the carboxylase.

The scattering curves of the dimer and tetramer of PEPc PCC 7002 showed the best fit with the available structures of PEPc from *E. coli* (Supplementary Information, Table S1). These two prokaryotes have similar C3 metabolism and the enzymes display 31% sequence identity, very slightly higher than the sequence identity of 30–31% between PEPc PCC 7002 and sequences from plants (Maize, *Flaveria, Arabidopsis*, Supplementary Information, Fig. S3). The analysis shows that all PEPc dimer structures, except the one from *Clostridium* where the protein is significantly smaller, are very similar in shape but that the inter subunit packing in the tetramer shows larger variation resulting in poorer fits to the SAXS data. Homology modelling of PEPc PCC 7002 based on available crystal structures (data not shown) did not generate a model with better fit to the SAXS profiles. This indicates that the main difference between the solution structure of the PEPc PCC 7002 tetramer and the crystal structures of *E. coli* PEPc may be in the inter-subunit packing. Future research will elucidate the structural details of the PEPc tetramer from cyanobacteria.

## Methods

### Construction of plasmid

To amplify *pepc*, gene encoding PEPc, from *Synechococcus* PCC 7002, genomic DNA was used as template together with Phusion High-Fidelity Hot Spot II DNA polymerase (Finnzymes) and primers for the gene with overhangs for restriction enzymes digestion (For-CTGAAGATCTAACCAAGTCATGCATCCCCC; Rev-GCATCTGCAGTCAACCCGTGTTCCGCAT). Obtained PCR product was purified (Gene JET Purification kit, Thermo Scientific) and digested by BglII and PstI fast restriction enzymes (Fermentas). The modified pET plasmid (pETBB) with a Strep-tag on the N-terminus and Bio brick assembly incorporated was digested by BamHI and PstI fast restriction enzymes. Both digested products (PCR and pETBB) were purified (DNA Clean & Concentrator-5, Zymo Research) and ligated using the Quick Ligation Kit (New England Biolabs) at room temperature for 15 min.

### Transformation of *E. coli* BL21

50 µL of thawed ice cold competent cells of *E. coli* BL21 were mixed with 5 µL of ligation mixture and incubated on ice for 30 min. After that, a heat shock was performed for 1 min at 42 °C before incubated on ice for 5 more min. 450 µL of Luria Broth (LB) media (room temperature) was added in the tube, mixed and incubated at 37 °C for 1 hour. Finally, the cells were centrifuged for 2 min at 13300 rpm and 450 µL of the supernatant was discarded. The cells were resuspended with the remaining supernatant and spread onto a LB agar place containing Kanamycin (Km) (50 µg · mL^−1^) before placed overnight at 37 °C.

Overnight colonies were used to run a PCR in order to verify incorporation of the genetic construct. Dream *Taq* DNA polymerase protocol (Fermentas) was used together with detailed primers (Section 2.1). Positive colonies were grown using LB media containing 50 µg · mL^−1^ Km and incubated shaking, at 37 °C, overnight. Next day, the plasmids were extracted from the cells using JET Plasmid Miniprep Kit (Thermo Scientific) and sequenced (Eurofins) in order to confirm that the correct plasmid, and DNA sequence, was successfully transformed into the cells.

### Cultivation of *E. coli* BL21

10 ml of overnight culture was inoculated in one litre of LB media supplemented with 5% of glucose and Km (50 µg·mL^−1^) (total 6 L) and placed at 37 °C under shaking conditions (150 rpm) until OD_600_ reached 0.4–0.6 (determined using UV-Visible Spectrophotometer 50 Bio). Then, the cells were cooled down to room temperature and induced with 0.1 mM of IPTG. Afterwards, the cells were grown overnight at 25 °C under constant shaking at 150 rpm. Next day, the cells were spun down at 5000 rpm, 10 min at 4 °C, and resuspended with TBS (100 mM Tris-HCl and 150 mM NaCl, pH 8.0) containing 25 mM MgCl_2_, pH 8.0, 1 mg of RNAse, 1 mg of DNAse and 0.6 mg·mL^−1^ of inhibitor protease cocktail (G-Biosciences). The cells were then broken using a sonicator (Chemical Instruments AB, Model CV33) at 60% amplification, with intervals of 15 sec and 10 sec, and kept on ice. The mixture was centrifuged at 15000 rpm for 1 h at 4 °C. Obtained supernatant was transferred into Falcon tubes and frozen using liquid nitrogen.

### Purification of PEPc PCC 7002

The supernatant was thawed on ice and then centrifuged at 15000 rpm for 15 min at 4 °C, while the column (Strep Tag HP-GE Healthcare) was connected to an ÄKTA system at 4 °C (GE-Healthcare). Then, the supernatant was filtered and loaded into the already equilibrated column (TBS + 25 mM MgCl_2_ pH 8.0) at a flow rate of 2 mL· min^−1^. When the protein was loaded into the column, a washing step was performed using TBS containing 25 mM MgCl_2_, pH 8.0 at 4 °C. The attached Strep tag protein was eluted from the column using TBS containing 25 mM MgCl_2_ pH 8.0 and 2.5 mM of desthiobiotin, concentrated using the Amicon Ultra-15 Centrifugal Filter Units MW 30 kDa, centrifuged at 5000 rpm for 20 min at 4 °C and frozen using liquid nitrogen. The concentration of the protein was measured using a UV-Visible Spectrophotometer (50 Bio) and the Lambert Law^38^.

### Size exclusion chromatography (SEC)

The purified protein in TBS buffer containing 25 mM MgCl_2_ and 2.5 mM desbiothin pH 8.0, was dialysed using the Slide-A-lyzer R Dialysis Cassette G2 (Thermo Scientific), to TBS buffer pH 8.0, changing the buffer three times every two hours before left overnight at 4 °C under constant stirring. The dialyzed protein was then aliquoted and frozen with liquid nitrogen.

The column used for the Size Exclusion Chromatography (SEC, Hi L oad 16/60 Superdex 200, GE-Healthcare) was equilibrated with indicated buffer depending on experi ment (Table 1) at 4 °C. 500 µg of purified protein was diluted (up to 570 µl (0.87 mg · ml^−1^) with the same buffer as the column was equilibrated with before incubated at room temperature for 30 min. The protein was then loaded in the column and eluted (0.8 mL·min^−1^) at 4 °C with the same buffer as the column was previously equilibrated with before the protein was diluted and incubated. When the different sizes of proteins were eluted, they were concentrated using the Amicon Ultra-15 Centrifugal Filter Units MW 30 kDa at 5000 rpm for 20 min at 4 °C and frozen with liquid nitrogen. The concentration of the proteins were measured as detailed above. The ratios of the tetramer:dimer were calculated using the peak values obtained on the chromatogram.

### SAXS

SAXS experiments were performed at beamline B21, Diamond Light Source, UK. 451 µg of dialysed PEPc PCC 7002 was incubated in either 400 µl of TBS containing 25 mM MgCl_2_ pH 8.0 or TBS pH 8.0 for 30 min at room temperature. Then, the protein was concentrated by centrifugation using a 30 kDa cut off concentrator (Amicon R Ultra). For SEC-SAXS, 50 µl protein at 6.5 or 0.87 mg · ml^−1^ concentration was using an Agilent 1200 HPLC system loaded to a 4.6 ml Shodex KW-403 column run at 0.16 ml · min^−1^ at room temperature in TBS or TBS with 25 mM MgCl_2_. For batch experiments, 25 µl of 0.2, 0.5, 1, 3 or 4.29 mg · ml^−1^ of protein were used in TBS pH 8.0. Data were recorded on an Eiger 4M detector with a fixed camera length of 4.014 m and 12.4 keV energy, allowing an angular q range of 0.0038–0.42 Å^−1^.

The SEC-SAXS data were buffer subtracted using buffer data frames in proximity to the peak using ScÅtter^39^ while batch data were buffer subtracted using Primus^40^. Data processing was performed using ScÅtter^39^ and Primus^40^ to obtain the radius of gyration (R_g_), the maximum particle dimension (D_max_), the excluded particle volume (Vp) and the pair distribution function (P(r)). In addition, Rg and MW were derived using the SAXS MoW server^41^. Theoretical scattering curves from PDB coordinates and their fits to the experimental scattering curves were calculated using the FOXS server^42^.

### Sequence alignment

Structure-guided multiple sequence alignment of PEPc PCC 7002 with all PEPcs in the PDB was done using the Expresso server^43^.

### *In vitro* PEPc activity measurements

The *in vitro* PEPc activity assays were performed as described by Codd and Stewart^44^ by adding 100 mM Tris pH 8.0 (except for the pH experiment where the pH varied from 6.5 to 9.0), 10 mM of MgCl_2_, 5 mM of NaHCO_3_, 0.15 mM of NADH, 5 mM of PEP and 8.25 units of malate dehydrogenase (MDH) from Heart Porcine (Merck). Then, the absorbance at 340 nm was measured (using a UV-Visible Spectrophotometer 50 Bio) and the reaction was activated by adding 0.348 µg of purified and SEC obtained PEPc PCC 7002 of the TBS (containing 25 mM MgCl_2_, pH 8.0) experiment tetrameric fraction (Mg_25, Table 1, Supplementary Information Fig. S1-B). The mixtures were incubated for 10 min at 35 °C (except for finding the optimal pH where 23 °C was used) in a temperature incubator and the absorbances were measured again. The slope obtained by the measurements before and after the reaction was used to calculate the PEPc activity, expressed as units · mg^−1^ of protein (where 1 unit represents 1 µmol of NAD^+^ formed · min^−1^). When the reaction was performed with 10 mM MgCl_2_, 5 mM PEP and no NaHCO_3_^−^ the enzyme showed some activity. The value obtained in the later reaction was subtracted from all the values obtained during the kinetics except for the pH experiment. In order to calculate the Km and Vmax, Hanes-Woolf plot was used. The activation energy was calculated by using the Arrhenius equation plotting the Vmaxs from 0 to 35 °C.

When the potential inhibitors or activators were tested (2 mM malate, 5 mM 2-oxoglutarate, 2 mM succinic acid, 5 mM glycine, 2 mM aspartic acid, 2 mM glutamine and 2 mM citric acid), the procedure for the activity assay was the previously described but the amount of substrates were lowered to 1 mM NaHCO_3_ and 1 mM PEP. In order to conclude if the substances tested were affecting the PEPc activity, T-Test were performed, with two tail distribution, two sample equal variance and p value 0.05.

## Supplementary information

Supplementary Information.
