# Supplementary material for: Oligomerization and characteristics of phosphoenolpyruvate carboxylase in Synechococcus PCC 7002
Source: Sci Rep. 2020 Feb 27;10:3607. doi: 10.1038/s41598-020-60249-2 (PMC7046716; doi:10.1038/s41598-020-60249-2)
Supplement: Supplementary file 1 — Supplementary Information. [file 41598_2020_60249_MOESM1_ESM.pdf]

Oligomerization and characteristics of phosphoenolpyruvate carboxylase in *Synechococcus* PCC 7002

Claudia Durall[a], Sandesh Kanchugal[b], Maria Selmer[b] and Peter Lindblad[a*]

SUPPLEMENTARY MATERIAL

Figure S1
Figure S2
Table S1

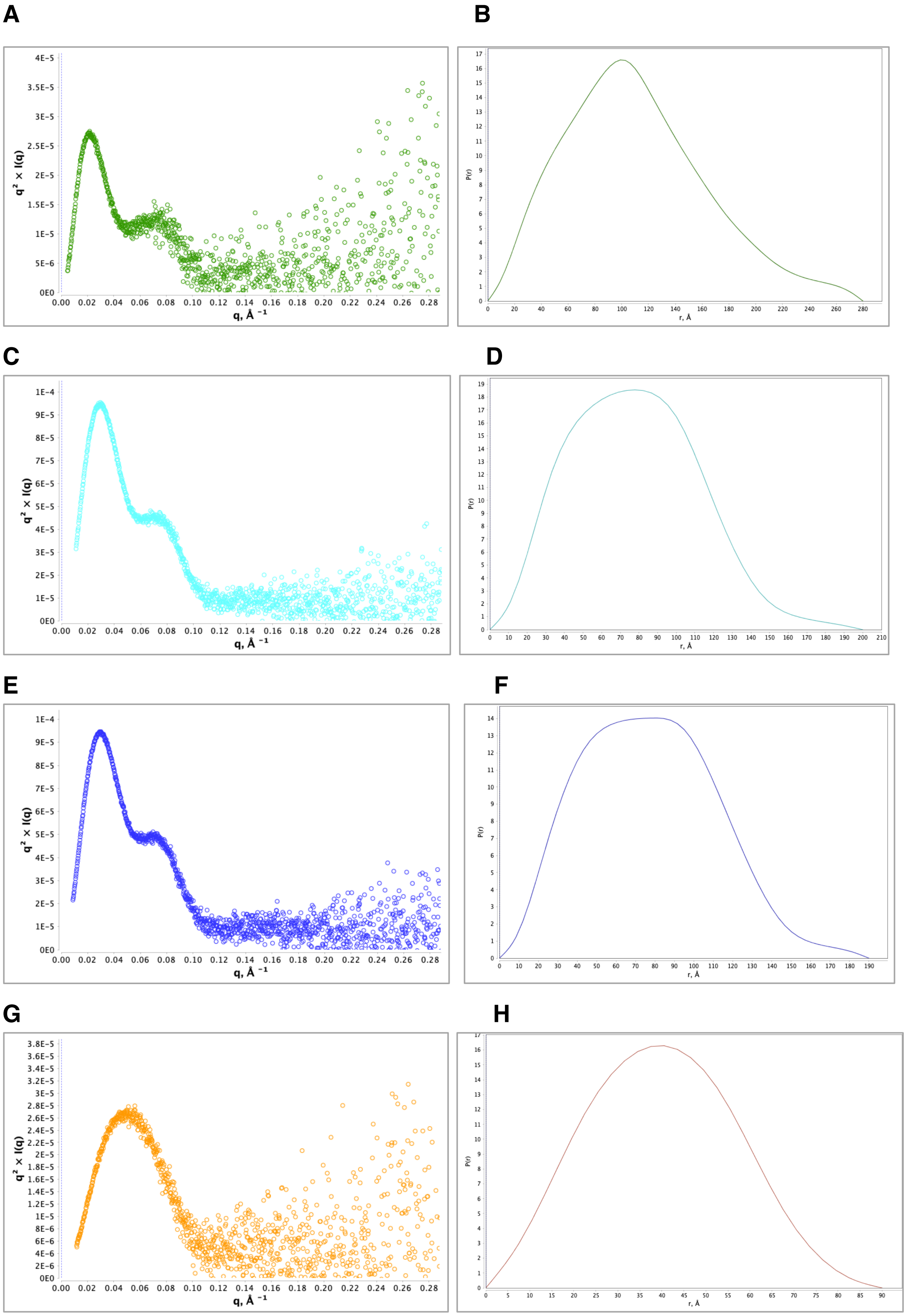
A
B
C
D
E
F
G
H
q² × I(q)
q, Å⁻¹
P(r)
r, Å

Figure S1. SEC-SAXS data analysis containing Kratky plots and Distance distribution P(r) for PEPc from Figure 8. The figures are generated from ScÅtter. **A-B**. Peak 1 in TBS with 25 mM $MgCl_2$ **C-D.** Peak 2 in TBS with 25 mM $MgCl_2$. **E-F.** Peak 2 in TBS. **G-H.** Peak 3 in TBS.

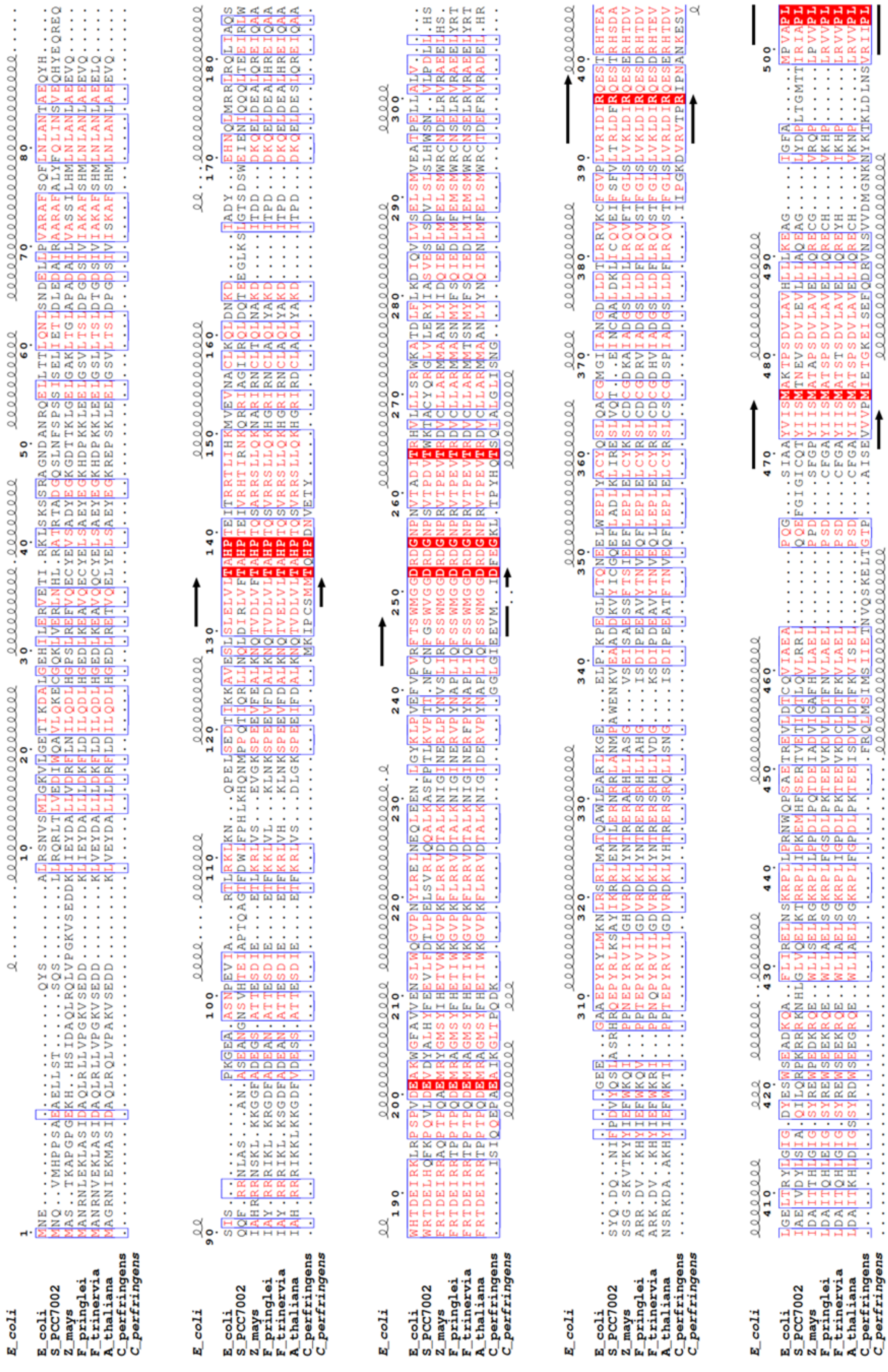

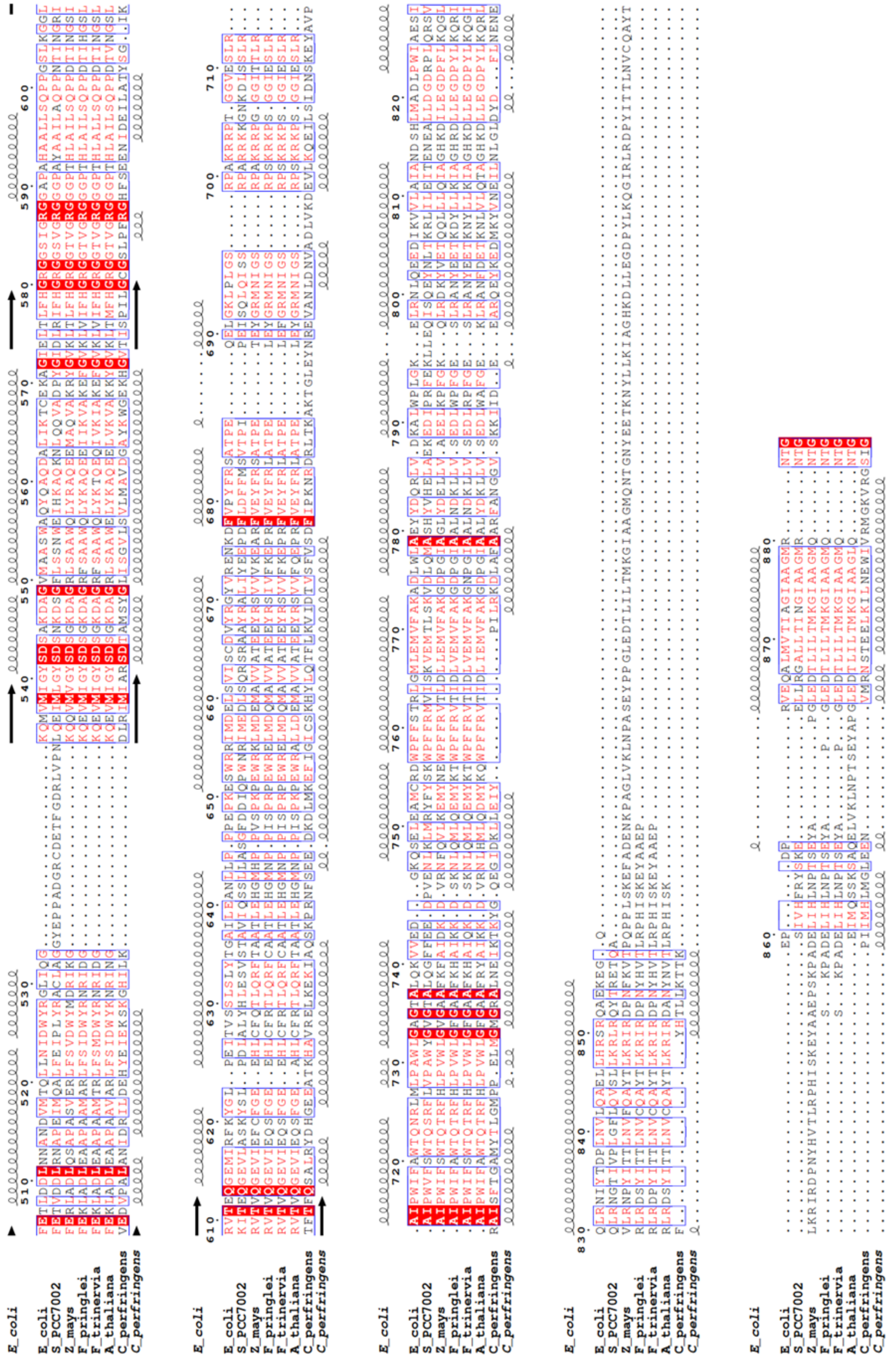

Figure S2. Multiple sequence alignment of PEPc PCC 7002 with PEPc variants with available structures. Above the alignment is shown secondary structure of *E. coli* PEPc from PDB 1FIY and below the alignment the secondary structure of *Clostridium perfringens* PEPc from PDB 3ODM.

Table S1. Comparison of experimental scattering curves with calculated scattering curves generated from available PEPc structures in the PDB using the FOXS server. Best fits for each peak are highlighted in green.

PDB	Reference	Organism	Ligand	Oligomeric state*	R_g (Å)	χ^2 dimer peak TBS	χ^2 tetramer peak TBS	χ^2 tetramer peak TBS + Mg^{2+}
1FIY	24	*E. coli*	Aspartic acid	Tetramer	54.7	35.7	5.97	5.03
				Dimer	40.2	1.48	581	499
1JQN	25	*E. coli*	Mn2+, DCDP, aspartic acid	Tetramer	54.8	36.3	5.99	5.63
				Dimer	40.5	1.47	523	453
1JQO	25	*Zea mays*	Sulfate	Tetramer	55.2	30.5	7.18	8.06
				Dimer	40.0	1.61	628	537
1QB4	45	*E. coli*	Mn2+, aspartic acid	Tetramer	54.8	35.8	5.56	5.39
				Dimer	40.2	1.47	594	510
3ODM	46	*Clostridium perfringens*	Malonate, gold cyanide	Tetramer	43.5	9.11	332	287
				Dimer	33.6	4.85	1253	1044
3ZGB	47	*Flaveria pringlei*	Sulfate, ethylene glycol, aspartic acid	Tetramer	49.3	26.5	103	82.8
				Dimer	40.0	1.55	628	537
3ZGE	47	*Flaveria trinervia*	Sulfate, ethylene glycol, aspartic acid	Tetramer	49.2	26.9	104	83.3
				Dimer	40.0	1.52	619	530
4BXC	48	*Flaveria trinervia*	Sulfate, glucose-6-phosphate, ethylene glycol, Tris	Tetramer	49.3	27.3	105	83.9
				Dimer	39.9	1.56	621	531
4BXH	48	*Flaveria trinervia*	Sulfate, ethylene glycol	Tetramer	49.3	27.7	103	83.0
				Dimer	40.0	1.56	623	532
5FDN	Un-published	*Arabidopsis thaliana*	Citrate, aspartic acid	Dimer#	39.9	1.55	634	542
5VYJ	49	*Zea mays*	Glycine, acetate	Tetramer	53.5	32.4	22.7	19.36
				Dimer	40.2	1.62	563	484

* Oligomers generated from crystal packing

No symmetric tetramer in crystal packing

REFERENCES

24. Kai, Y., Matsumura, H., Inoue, T., Terada, K., Nagara, Y., Yoshinaga, T., Kihara, A., Tsumura, K., Izui, K. (1999) Three-dimensional structure of phosphoenolpyruvate carboxylase: a proposed mechanism for allosteric inhibition. *Proc. Natl. Acad. Sci.* 96, 823-828.

25. Matsumura, H., Xie, Y., Shirakata, S., Inoue, T., Yoshinaga, T., Ueno, Y., Ueno Y, Izui K, Kai, Y. (2002) Crystal structures of C4 form maize and quaternary complex of *E. coli* phosphoenolpyruvate carboxylases. *Structure* 10, 1721-1730.

45. Matsumura, H., Terada, M., Shirakata, S., Inoue, T., Yoshinaga, T., Izui, K., Kai, Y. (1999) Plausible phosphoenolpyruvate binding site revealed by 2.6 Å structure of Mn^{2+}-bound phosphoenolpyruvate carboxylase from *Escherichia coli*. FEBS Lett. 458, 93-96.

46. Dharmarajan, L., Kraszewski, J. L., Mukhopadhyay, B., Dunten, P. W. (2011) Structure of an archaeal-type phosphoenolpyruvate carboxylase sensitive to inhibition by aspartate. Proteins: *Struct. Funct. Bioinf.* 79, 1820-1829.

47. Paulus, J. K., Schlieper, D., Groth, G. (2013) Greater efficiency of photosynthetic carbon fixation due to single amino-acid substitution. *Nat. Commun*. 4, 1518.

48. Schlieper, D., Förster, K., Paulus, J. K., Groth, G. (2014) Resolving the activation site of positive regulators in plant phosphoenolpyruvate carboxylase. *Mol. Plant* 7, 437-440.

49. González-Segura, L., Mújica-Jiménez, C., Juárez-Díaz, J. A., Gümez-Toro, R., Martinez-Castilla, L. P., Muñoz-Clares, R. A. (2018) Identification of the allosteric site for neutral amino acids in the maize C4 isozyme of phosphoenolpyruvate carboxylase: The critical role of Ser-100. *J. Biol. Chem*. 293, 9945-9957.
